# Influenzanet: Citizens Among 10 Countries Collaborating to Monitor Influenza in Europe

**DOI:** 10.2196/publichealth.7429

**Published:** 2017-09-19

**Authors:** Carl E Koppeschaar, Vittoria Colizza, Caroline Guerrisi, Clément Turbelin, Jim Duggan, W John Edmunds, Charlotte Kjelsø, Ricardo Mexia, Yamir Moreno, Sandro Meloni, Daniela Paolotti, Daniela Perrotta, Edward van Straten, Ana O Franco

**Affiliations:** ^1^ De Grote Griepmeting Science in Action BV Amsterdam Netherlands; ^2^ UPMC Univ Paris 06, Institut National de la Santé et de la Recherche Médicale (INSERM), Institut Pierre Louis d’épidémiologie et de Santé Publique (IPLESP UMRS 1136), Sorbonne Universités Paris France; ^3^ School of Engineering and Informatics National University of Ireland Galway Ireland; ^4^ Faculty of Epidemiology and Population Health London School of Hygiene and Tropical Medicine London United Kingdom; ^5^ Statens Serum Institute København Denmark; ^6^ Instituto Nacional de Saúde Doutor Ricardo Jorge Lisbon Portugal; ^7^ Institute for Biocomputation and Physics of Complex Systems Department of Theoretical Physics University of Zaragoza Zaragoza Spain; ^8^ Institute for Scientific Interchange Foundation Torino Italy; ^9^ Public Health Agency of Sweden Solna Sweden; ^10^ Instituto Gulbenkian de Ciência Oeiras Portugal

**Keywords:** influenza, surveillance, Internet, vaccination, Europe

## Abstract

**Background:**

The wide availability of the Internet and the growth of digital communication technologies have become an important tool for epidemiological studies and health surveillance. Influenzanet is a participatory surveillance system monitoring the incidence of influenza-like illness (ILI) in Europe since 2003. It is based on data provided by volunteers who self-report their symptoms via the Internet throughout the influenza season and currently involves 10 countries.

**Objective:**

In this paper, we describe the Influenzanet system and provide an overview of results from several analyses that have been performed with the collected data, which include participant representativeness analyses, data validation (comparing ILI incidence rates between Influenzanet and sentinel medical practice networks), identification of ILI risk factors, and influenza vaccine effectiveness (VE) studies previously published. Additionally, we present new VE analyses for the Netherlands, stratified by age and chronic illness and offer suggestions for further work and considerations on the continuity and sustainability of the participatory system.

**Methods:**

Influenzanet comprises country-specific websites where residents can register to become volunteers to support influenza surveillance and have access to influenza-related information. Participants are recruited through different communication channels. Following registration, volunteers submit an intake questionnaire with their postal code and sociodemographic and medical characteristics, after which they are invited to report their symptoms via a weekly electronic newsletter reminder. Several thousands of participants have been engaged yearly in Influenzanet, with over 36,000 volunteers in the 2015-16 season alone.

**Results:**

In summary, for some traits and in some countries (eg, influenza vaccination rates in the Netherlands), Influenzanet participants were representative of the general population. However, for other traits, they were not (eg, participants underrepresent the youngest and oldest age groups in 7 countries). The incidence of ILI in Influenzanet was found to be closely correlated although quantitatively higher than that obtained by the sentinel medical practice networks. Various risk factors for acquiring an ILI infection were identified. The VE studies performed with Influenzanet data suggest that this surveillance system could develop into a complementary tool to measure the effectiveness of the influenza vaccine, eventually in real time.

**Conclusions:**

Results from these analyses illustrate that Influenzanet has developed into a fast and flexible monitoring system that can complement the traditional influenza surveillance performed by sentinel medical practices. The uniformity of Influenzanet allows for direct comparison of ILI rates between countries. It also has the important advantage of yielding individual data, which can be used to identify risk factors. The way in which the Influenzanet system is constructed allows the collection of data that could be extended beyond those of ILI cases to monitor pandemic influenza and other common or emerging diseases.

## Introduction

Influenza is a global public health problem—whether seasonal, zoonotic, or pandemic—causing high general practice consultation rates, increased hospital admissions, excess deaths, and high absenteeism in schools and workplaces, including in health workers. Its high socioeconomic impact and burden is not just limited to the industrialized world but extends to low- and middle-income countries, encompassing multiple dimensions such as direct costs to the health service and households and indirect costs because of productivity losses, as well as broadly affecting the overall economy [1].

Influenzanet [2] is a participatory monitoring system for influenza-like illness (ILI) based on data reported by Internet users among the general population who volunteer as participants. It was initially conceived to make scientific information accessible to a broad public and to promote students’ enthusiasm for science [3,4]. It was first launched in the Netherlands and Belgium as “The Great Influenza Survey” (De Grote Griepmeting [5]) in the 2003-04 influenza season. In 2005, Portugal joined (Gripenet [6]). Subsequently, the system was adopted by Italy (Influweb [7]) in 2008, the United Kingdom (Flusurvey [8]) in 2009, Sweden (Hälsorapport [9]) in 2011, France (Grippenet [10]) and Spain (Gripenet.es [11]) in 2012, and Ireland (Flusurvey.ie [12]) and Denmark (Influmeter [13]) in 2013. Switzerland joined in December 2016; however, at the moment of the writing of the paper, there were not enough data to be included in the analysis.

**Figure 1:**
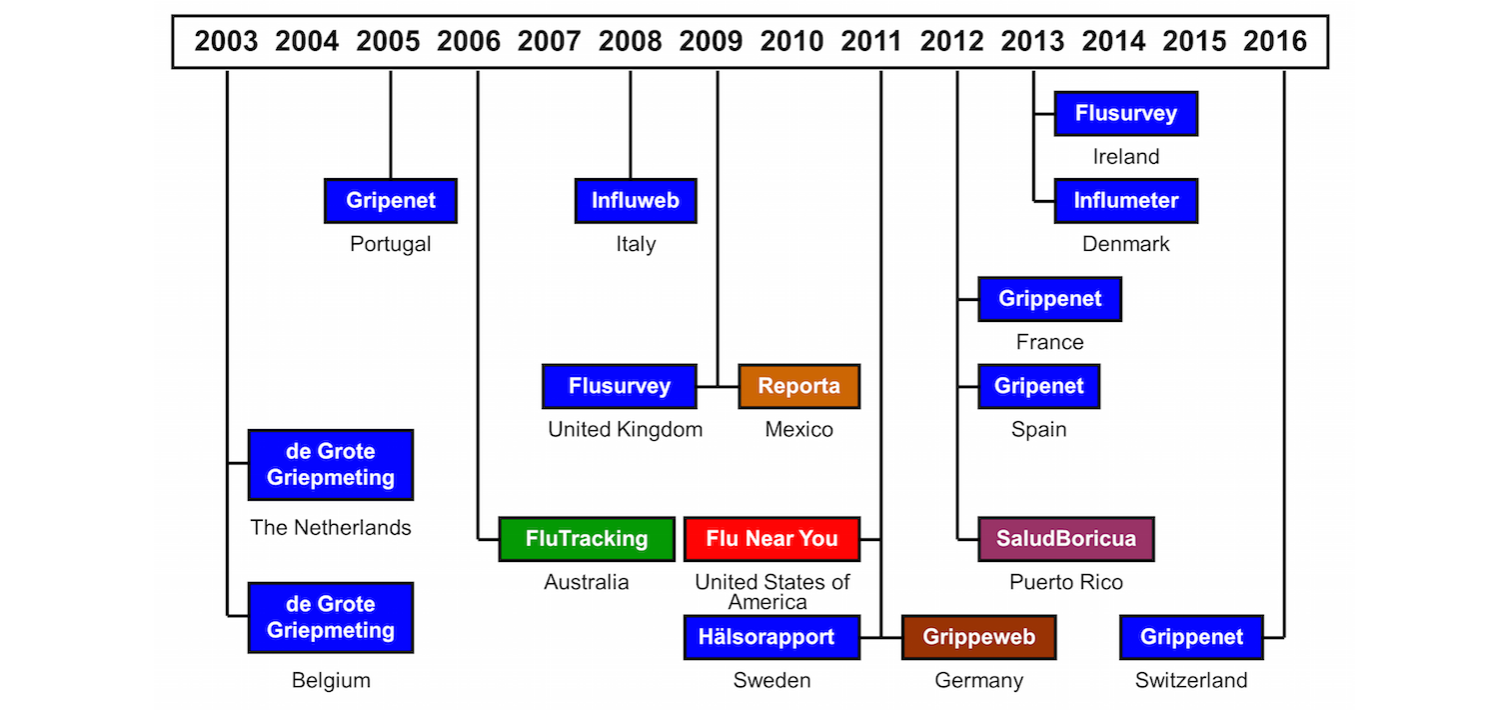
Timeline of Influenzanet (in blue) and other participatory surveillance systems for influenza-like illness.

In 2009, the Influenzanet consortium was established to foster collaboration and pool resources toward using this uniform system of participatory ILI surveillance across Europe. Hereafter, we will refer to the system in each country as Influenzanet, instead of designating it by the actual name by which the system is known in each country. The Portuguese team also helped to introduce the system in Latin America, namely in Mexico (Reporta [14]) and supported the development of a similar system in Brazil but focused on dengue instead (Dengue na Web [15]). Similar systems were independently implemented in Australia (Flu Tracking [16]), the United States (Flu Near You [17]), and Germany (GrippeWeb [18]). Additionally, Salud Boricua [19] was launched in Puerto Rico, targeting 3 different acute febrile illnesses, which included influenza, dengue, and leptospirosis. Figure 1 (updated from [20]) shows a timeline of the launch date of the participatory surveillance systems for ILI in Europe and worldwide.

Here, we describe the Influenzanet participatory surveillance system and provide an overview of the results obtained from different analyses performed with the data, including representativeness analyses, data validation (comparing ILI incidence rates between Influenzanet and sentinel medical practice networks), identification of ILI risk factors, and influenza vaccine effectiveness (VE) studies previously published. Additionally, we present new VE analyses for the Netherlands, stratified by age and chronic illness, and offer suggestions for further work and considerations on the continuity and sustainability of the participatory system.

## Methods

### Influenzanet Data Collection

Any resident of a participating country can register on its national website by completing a simple Web-based intake questionnaire containing various sociodemographic, medical, and behavioral questions, in addition to the questions about postal code of residence and workplace (see Multimedia Appendix 1). Once registered, participants receive a weekly email newsletter with a reminder to complete a short symptoms questionnaire. In this questionnaire, participants are asked to report any symptoms that they have experienced since their previous visit to the Influenzanet website. If symptoms are reported, participants are asked to provide further information, including the date of onset, whether these led to a change of behavior (eg, missing school or work or taking medicines), and whether the participant visited a medical service, and if so, the outcome of the consultation. The system allows participants to also report for other members of their household to foster data collection for children and elderly people. On the basis of a unique user identifier, participants can be followed over multiple seasons and are urged to update changes in sociodemographic, medical, behavioral, residential, and workplace information every season.

### Participation Rates

**Figure 2:**
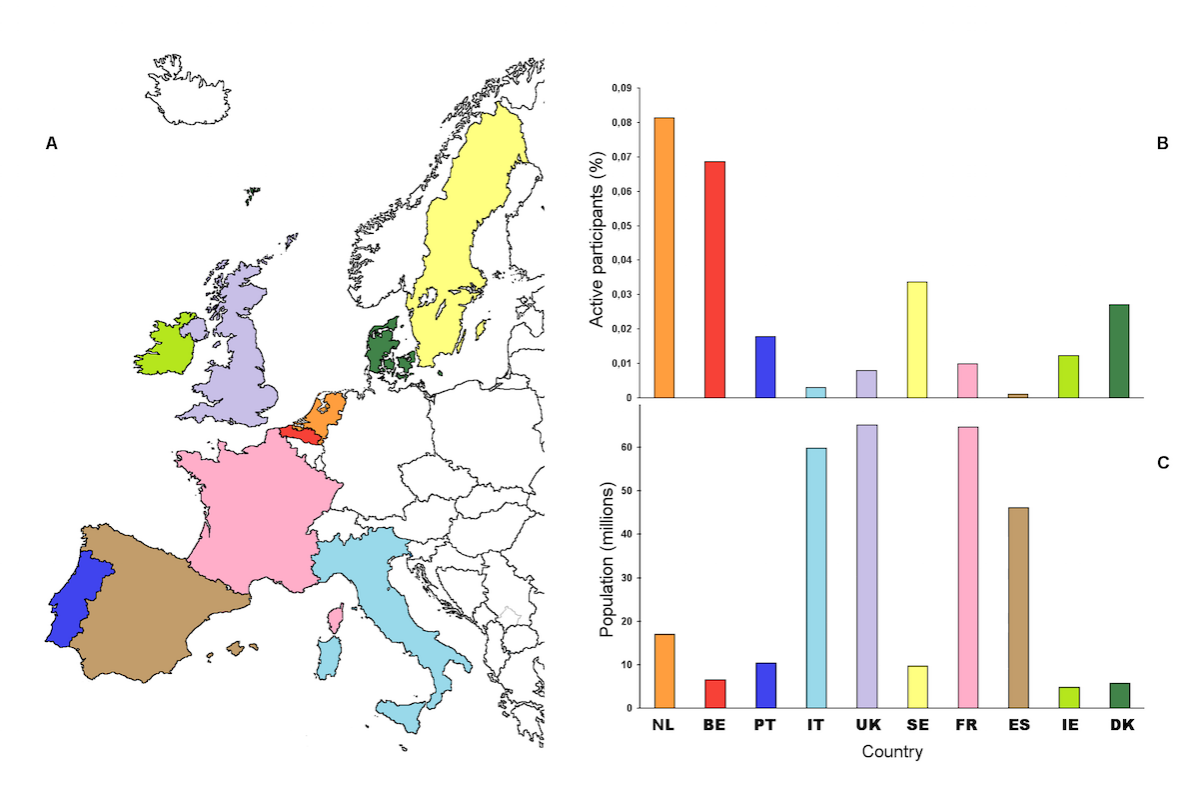
Participation rates across the 10 Influenzanet countries. Percentage of Influenzanet active participants (B) among each country’s total population (C). Source: Influenzanet 2015-16 data for all countries, except for Sweden where data are for 2013-14; Country population per January 1, 2016 (Sweden per January 1, 2014)—the Netherlands (NL): 17 million (M), Belgium (BE): 7M, Portugal (PT): 10M; Italy (IT): 60M; The United Kingdom (UK): 65M; Sweden (SE): 10M; France (FR): 65M; Spain (ES): 46M; Ireland (IE): 5M; and Denmark (DK): 6M.

Several thousands of participants have been engaged yearly in Influenzanet, with over 36,000 volunteers in the last season of 2015-16. On the basis of the 2015-16 season, the Netherlands had the highest number of participants who completed at least 3 symptoms questionnaires (13,821 participants corresponding to 0.08% of the country’s population), followed by France (6413; 0.01%), the United Kingdom (5134; 0.01%), Belgium (Dutch-speaking region: 4559; 0.07%), Sweden (3245; 0.03%), Portugal (1840; 0.02%), Italy (1822; 0.003%), Denmark (1541; 0.03%), Ireland (575; 0.01%), and Spain (487; 0.001%). Data for Sweden are for the 2013-14 season, as after that Sweden started using Influenzanet through invitation only to improve the representativeness of the monitored sample and compare it with the previous seasons. Figure 2 compares the participation rates across countries versus the country’s population [21].

The number of participants has been relatively stable over the years for the countries that first started (after having increased during their first seasons) and is still increasing for the countries that joined after 2011. For example, of all participants who completed at least 3 symptoms questionnaires during a season (hereafter referred as active participants) analyzed from 2003-04 to 2012-13, on average in the Netherlands, 76% (Standard deviation [SD] 8; N=16,481) participated again in the following season, with 69% (SD 12; N=1894) in Portugal, whereas Belgium and Italy had values within that range [22]. During the influenza season from November 2013 until May 2014 (29 weeks of survey), 73% (9479/12,985) of the participants in the Netherlands completed the survey more than 20 times; 68% (7964/11,758) of the completed surveys were less than 9 days apart, reflecting a high level of engagement of the participants with the system [4].

### Ethical Approval

In all the participating countries, Influenzanet studies were conducted in agreement with national regulations on privacy and data collection and treatment. Informed consent was obtained from individuals who participated in the studies enabling the collection, storage, treatment, and publication of data in anonymized, processed, and aggregated forms for scientific purposes. The Grote Griepmeting study is carried out according to the Dutch legislation on privacy, and the privacy regulation of the studies was approved by the Dutch Data Protection Authority. In Portugal, the Gripenet project was approved by the Ethics Committee of the Instituto Gulbenkian de Ciência, and the Portuguese Data Protection Commission approved the Gripenet study (Authorization Number 2868). In the United Kingdom, the Flusurvey was approved by the London School of Hygiene and Tropical Medicine Ethics Committee (Application Number 5530). In Sweden, the Influensaskoll study was approved by the Stockholm Regional Ethical Review Board (Dnr. 2011/387-31/4). In France, the Grippenet study was approved by the French Advisory Committee for research on information treatment in the field of health (ie, CCTIRS, authorization 11.565) and by the French National Commission on Informatics and Liberty (authorization DR-2012-024).

## Results

### Participant Representativeness

In Web-based surveys, the nonrepresentative nature of the Internet-using population can result in a selection bias [3]. In addition, people who do not experience any ILI symptoms may not consider themselves suitable for participation. Accordingly, representativeness analyses have been performed at various stages during the activity of Influenzanet to compare the demographic and health characteristics of the participants with those in the country's overall population.

In work from 2006, it was shown that the demographic and health characteristics of the participants in the Netherlands were remarkably similar with those observed by the National Information Network of General Practicioners (at that time Landelijk Informatie Netwerk Huisartsenzorg abbreviated as LINH; [3]). Namely, striking similarities were found between Dutch Influenzanet participants (N=13,000) and the population observed by the general practitioners (GPs) in LINH (N=255,000) with regard to the prevalence of asthma (6.9% in Influenzanet, n=918 vs 6.4% in LINH, n=6320) and influenza vaccination rates and, to a lesser degree, for diabetes (2.4% in Influenzanet, n=319 vs 3.5% in LINH, n=8925; *P*<.005). The vaccination rates in patients with asthma, diabetes, and persons older than 65 years were 68% (n=9044), 85% (n=11,305), and 85% (n=11,305), respectively, among Dutch Influenzanet participants, whereas the corresponding percentages in the LINH population were 73% (191,250), 85% (216,750), and 87% (221,850). Similar results were obtained for Belgium [23] and the United Kingdom [24] in terms of risk group status.

In 2011-12, Influenzanet launched a standardized common framework for data collection. A study of representativeness was then extended to all participating countries (7 at that time) to assess the representativeness of the sample in terms of a set of demographic, geographic, socioeconomic, and health indicators [25]. The Influenzanet population was not representative of the general population in terms of age distribution, underrepresenting the youngest and the oldest age groups. However, all age classes were represented. The gender imbalance differed between countries, although higher female participation occurred in most countries (the Netherlands, the United Kingdom, Sweden, and France). Differences between gender-specific information-seeking behavior (more prominent in women) and Internet usage (with higher rates in male populations) may have been at the origin of these gender imbalances. For instance, the countries with higher Internet usage by males were also the countries either having a larger prevalence of male Influenzanet participants (Belgium and Italy) or displaying similar participation of males and females (Portugal).

In the aforementioned 2011-12 representativeness study [25], smokers were underrepresented in the majority of countries, as were individuals with diabetes; the representativeness of asthma prevalence and influenza vaccination coverage for ≥65 years individuals in 2 successive seasons (2010-11 and 2011-12) varied between countries. Additionally, participants from most countries were found to be more frequently employed than the general population, except in the Netherlands where the contrary was observed, and in the United Kingdom where no significant difference was found. Participants also tended to have a higher education level than the general population, as shown by results from the 3 countries where such data were available to compare with Influenzanet data (France, Portugal, and Sweden; [25,26]).

Quantifying these biases allows them to be taken into account in future analyses of Influenzanet epidemiological studies.

### Influenzanet Versus Traditional ILI Surveillance

Influenza surveillance in Europe is traditionally pooled by the European Influenza Surveillance Network (EISN), which combines epidemiological and virological surveillance of influenza. The EISN network includes a set of sentinel GPs in each country who collect information from patients reporting symptoms of ILI. The sentinel GPs report the aggregated number of ILI consultations, by age group, to the EISN via The European Surveillance System (TESSy) database, based on which the EISN calculates the ILI rates. A sample of these patients is also tested for virological confirmation of influenza. The sentinel GPs usually represent 1% to 5% of the GPs working in the country or region [27].

The EISN is coordinated by the European Centre for Disease Control and Prevention (ECDC) since 2008 and participates in the wider World Health Organization (WHO) Regional Office for European Region influenza network and in the WHO Global Influenza Surveillance and Response System. Since 2014, the ECDC and WHO/Europe have a single joint Web-based bulletin called “Flu News Europe” [28].

The incidence of ILI among Influenzanet participants is determined in near real time using a syndromic case definition. From season 2011-12 onwards, all Influenzanet countries apply the case definition for ILI recommended by the ECDC when reporting feedback to participants on whether their reported symptoms might be due to ILI. Additionally, as the participants’ individual symptoms data are available, this enables exploring different case definitions when analyzing the data (see Textbox 1). Graphic representation of the results is dynamically updated on the Influenzanet website.

Previous studies have established a positive correlation between the incidence of ILI determined by Influenzanet and that estimated through the clinical surveillance by sentinel GPs [3,22,23,29-33]. Although there is an approximately parallel rise, peak and decline of ILI activity between the Influenzanet and EISN epidemic curves, the incidence values obtained by Influenzanet are quantitatively higher than those collated by ECDC. For instance, Influenzanet ILI incidence rates in the Netherlands were found to be 5 to 10 times higher during the winters of 2010-11 to 2015-16 (when restricted to the period of virological influenza confirmation by the Dutch Sentinel Practice Network). Specifically, the percentage of ILI cases in the Dutch influenzanet volunteers ranged from 6% (616/10,803) to 14% (1532/11,034) during an observation period of 22 weeks, whereas the Dutch Sentinel Practice Network rates for the same period ranged from 0.8% to 2% (840 to 2220 per 100,000 patients) (unpublished data). Higher incidence rates in Influenzanet versus sentinel surveillance also occur in other participating countries, although the magnitude of the difference varies by country [22,30,32,33].

The greater magnitude of the incidence rates estimated by Influenzanet versus sentinel surveillance networks might possibly be partially explained by health care-seeking behavior, as this differs across countries. People may not seek medical care for a variety of reasons such as disease severity or sociodemographic differences and thus not be accounted as ILI cases by the traditional sentinel surveillance system [22].

Influenzanet allows estimating the fraction of the population with symptoms that seeks health care services, as this is a follow-up question asked to participants in the symptoms questionnaire. The data have shown that this fraction varies greatly by country [22], being also dependent on the severity of symptoms (and therefore, on the ILI case definition used [33,34]) and the season [22,34].

**Influenza-like illness case definitions.**

The following case definition for influenza-like illness (ILI) is recommended by the European Centre for Disease Control and Prevention (ILI^ecdc^). From season 2011-12 onwards, all countries participating in Influenzanet use the same questionnaires and apply this case definition when reporting feedback to participants on whether their reported symptoms might be due to ILI:

- Sudden onset of symptoms;
- AND at least 1 of the following systemic symptoms: Fever or feverishness (chills), Malaise, Headache, Muscle pain;
- AND at least 1 of these respiratory symptoms: Cough, Sore throat, Shortness of breath.

During the first seasons of Influenzanet, the questionnaire did not include some of the symptoms above; additionally, participants could only report fever if they measured their temperature. Therefore, the ILI^ecdc^ definition could not be applied. To overcome this and to allow comparing data across seasons, the following case definition was developed (ILI^hist^, for historic reasons):

- Sudden onset of symptoms;
- AND Fever (≥38°C temperature);
- AND at least 1 of these systemic symptoms: Headache, Muscle pain;
- AND at least 1 of these respiratory symptoms: Cough, Sore throat

ILI^ecdc^ has a higher sensitivity, because more participants with influenza will fit the definition. Conversely, ILI^hist^ has a higher specificity, since fewer participants who do not have influenza will fit the definition.

The ILI^hist^ case definition was first used in the Netherlands and Belgium in 2003 to closely match the Dutch general practitioners’ ILI case definition (sudden onset of symptoms with a prodromal phase of an already existing nonsickening respiratory infection of at the most 3 to 4 days; and fever (≥38°C temperature); and at least one of the following symptoms: cough, runny nose, sore throat, frontal headache, retrosternal pain, or muscle pain).

In addition to these, multiple case definitions can be used within the Influenzanet system to analyze the symptoms data provided by the participants.

The percentage of participants with ILI who sought medical care was shown to be lower in northern Europe (except Belgium) than in southern Europe [22]. For instance, in the 2013-14 season, Danish Influenzanet data have demonstrated that the fraction of Danish participants with ILI who visited a GP ranged between 16% (31/192), 22% (92/413), and 34% (33/97), when considering the ILI case definition used by Danish GPs, ECDC, or the alternative ILI^hist^ definition, respectively ([33], complemented by Influenzanet unpublished data). In Belgium, a higher fraction of volunteers with ILI reported visiting a health care professional (71%, 112/158 ILI^hist^), possibly because according to the Belgian law, an employer can require from its employee a medicaI statement within 24 hours to justify work absenteeism ([22], complemented by Influenzanet unpublished data).

Among the Influenzanet volunteers who did seek medical care, in southern Europe (France, Italy, Portugal, and Spain) and Belgium, the participants reported to generally visit a GP within 1 to 2 days after the onset of ILI symptoms, whereas in northern Europe (Sweden, the United Kingdom, the Netherlands, Denmark) with the exception of Belgium, participants generally sought medical care only 5 to 7 days after the onset of symptoms [22]. In countries where participants wait longer before seeking medical care, many ILI cases may no longer feel sufficiently ill to warrant a visit to a health care professional and therefore are not accounted as ILI cases by the traditional sentinel surveillance system [22,30].

This variation across countries in the rates of seeking medical care is one of the reasons why ILI incidence reported by ECDC cannot be compared directly between countries. Another reason is the disparities in ILI case definitions used by GPs in different countries. For example, many national surveillance systems do not apply the ILI definition recommended by ECDC, where fever is not mandatory but instead apply an ILI case definition that does require fever, especially to distinguish between an influenza infection and a common cold [22].

Estimates of disease burden can be informative for public health policy decisions regarding the prioritization of interventions and preventive measures. As the traditional health care-based surveillance tends to underestimate the true burden of disease in the population, Influenzanet can be used as a supplemental data source to obtain a more comprehensive estimate of the true disease burden [35]. It could also target the economic burden, including direct costs through health care as well as the indirect socioeconomic costs (school and work absenteeism); it could additionally contribute to estimate the cost-effectiveness of vaccination.

### Identification of Risk Factors

The primary risk factor for acquiring an ILI infection is having direct or indirect contact with an infectious person. Analyses of individual-level data provided by the Influenzanet volunteers in the Netherlands, Belgium, Portugal, and Italy allowed identifying the following factors as additional independent predictors of increased risk of having at least one ILI episode during an influenza season [22]: having a chronic disease (asthma, diabetes, heart disease, and/or an immunocompromising condition), living with at least 1 child, belonging to a younger age group (<18 years of age), having one or more allergies (hay fever, dust mite allergy and allergy to cats and/or dogs), and being a smoker.

Seniors are generally considered a risk group for influenza, not because of a higher probability of infection but because of their greater risk for complications and increase in expected mortality [36,37]. In the Influenzanet study [22], the risk of ILI among participants over the age of 65 years was smaller than in the other age groups. This was not because of the higher uptake of influenza vaccine in seniors, as the risk factor analysis accounted for that factor by including vaccination status as a separate covariate in the multivariate model. The reduced risk among seniors may possibly be attributed to immunity from past exposures (ie, either from prior influenza infections and/or vaccinations). Immune responses of older adults are often markedly stronger than those of younger individuals for some influenza strains (eg, for A/H1N1 that circulated between 1918 and 1957 and that was reintroduced in 1976 and with a pandemic subtype in 2009) but are more similar for others (eg, A/H3N2 that has circulated since 1968; [38]). Alternatively, or additionally, the reduced risk among seniors may possibly also be because of a smaller contact rate with infectious individuals.

A small risk reduction was also observed in Influenzanet participants who practiced more than 1 hour of sports per week. Finally, public transportation did not appear to increase the risk of developing ILI relative to driving a car, riding a bicycle, or walking as a primary mode of transportation [22]. The results of these risk factor analyses have been shown to be consistent across all Influenzanet countries [39].

The identification of ILI risk factors is one of two main ways that ILI surveillance data have been used to gain a better understanding of ILI control and prevention; the evaluation of intervention effectiveness is another, as discussed in the next section.

### Influenza Vaccine Effectiveness

Vaccination to prevent influenza is particularly important for people who are at a higher risk of developing serious complications if they get sick with influenza. According to WHO, the recommended risk groups for vaccination are as follows: all people ≥6 months of age with a chronic disease, children aged 6 to 59 months, pregnant women, residents of long-term care facilities, health care workers, and elderly (often defined as aged ≥65 years, but defined as aged ≥60 years in the Netherlands; [37]). The ability of an influenza vaccine to protect someone depends not only on the age and health status of the person getting the vaccine but also on the similarity or “match” between the virus strains in the vaccine and those in circulation. Effectiveness against ILI is therefore expected to be lower for influenza, as the influenza vaccine targets specifically the influenza virus and not other ILI. According to a large meta-analysis of 90 reports containing 116 datasets of randomized or quasi-randomized studies of VE in healthy adults, the overall effectiveness of inactivated parenteral influenza vaccines was estimated to be 16% (95% CI 5-25) against ILI and 60% (95% CI 53-66) against confirmed influenza [40].

Ideally, large-scale randomized controlled trials should be undertaken to assess vaccine efficiency, but it is impractical to conduct them every year. Also, because of global recommendations for influenza vaccination, placebo-controlled trials that could clarify the effects of influenza vaccines in individuals are no longer considered possible on ethical grounds [41]. For these reasons, most data on influenza VE come from observational studies.

The I-MOVE (Influenza-Monitoring Vaccine Effectiveness) network [42] aims at measuring influenza VE in Europe and has been operating since 2007, coordinated by ECDC. Eight study sites (Germany, Hungary, Ireland, Italy, Poland, Portugal, Romania, and Spain) participated in the test-negative 2014-15 multicenter case–control study [43]. The methods are based on the ECDC generic case–control study protocol [44]. Participating GPs interviewed (collecting clinical and epidemiological information) and collected nasopharyngeal specimens from patients consulting for ILI aged ≥60 years (Germany, Poland, and 3 regions in Spain), or ≥65 years (Hungary, Ireland, Italy, Portugal, Romania, and 3 regions in Spain), and from a systematic sample of ILI patients in the other age groups. Only patients who presented to the GPs more than 14 days after the start of the national vaccination campaigns and who met the ECDC ILI case definition, and who had not received antivirals before swabbing, were swabbed within 7 days of symptom onset. For the 2014-15 season, the overall VE against influenza A(H3N2) was 14.4% (95% CI −6.3 to 31.0), against A(H1N1)pdm09 was 54.2% (95% CI 31.2-69.6), and against B was 48.0% (95% CI 28.9-61.9).

Because Influenzanet also collects data on whether participants have been vaccinated for influenza, it allows measurement of ILI incidence in the self-reporting cohorts of vaccinated and unvaccinated participants, and therefore, the system could potentially be used as a complementary tool to measure the effectiveness of the influenza vaccine against ILI close to real time [45].

Several of the Influenzanet national project teams have carried out studies to assess VE among participants during specific years. For example, UK Influenzanet data were used to estimate the effectiveness of the influenza vaccine in the postpandemic influenza season of 2010-11 [46]. In that study, vaccination for seasonal influenza in combination with the vaccination against the pandemic influenza the previous year was associated with reduced ILI incidence, with an estimated VE of 52% (95% CI 27-68). It was also associated with reduced absenteeism, especially for those between 25 and 64 years of age, with 4.1% of the vaccinated participants reporting taking time off work because of symptoms, compared with 11.6% of the unvaccinated persons (*P*<.001). Furthermore, vaccinated absentees were away from work for a significantly shorter period of time compared with the unvaccinated persons.

In France, the effectiveness of the 2012-13 influenza vaccine against ILI (defined by cough and fever ≥38^o^C in that study) was estimated as 49% (95% CI 20-67; *P*<.001) for the overall population and 32% (95% CI 0-58; *P*=.10) for the population at risk of developing influenza-related complications, based on data from Influenzanet participants in that season [47].

In the Netherlands, between 2003-04 and 2012-13, a reduction in ILI among vaccinated Influenzanet participants was estimated in 4 seasons (2007-08, 2008-09, 2010-11, and 2012-13), whereas in the other 6 seasons no statistically significant effect was observed [22]. The VE for all participants varied between 33% (95% CI 22-42) in 2010-11 and −10% (95% CI −28 to 6) in 2004-05. In addition to 2004-05, a negative although likewise nonstatistically significant VE was also estimated for 2003-04, both being seasons with a poor vaccine match with the circulating influenza virus strains [48].

### Additional VE Analyses

There are a few important considerations when using Influenzanet data as a complementary tool to estimate the effectiveness of the influenza vaccine. The influenza vaccine only protects against the influenza virus, but ILI may be caused by other infections. Additionally, vaccinated and unvaccinated participants cannot be compared directly, as participants who decide to take the vaccination may do so because they belong to a risk group, for instance, those with a chronic disease or those of older age; thus, differences in ILI rate between vaccinated and unvaccinated participants can be because of either the vaccine or an a priori difference between both groups [32]. Finally, influenza infections may develop asymptomatically.

**Figure 3:**
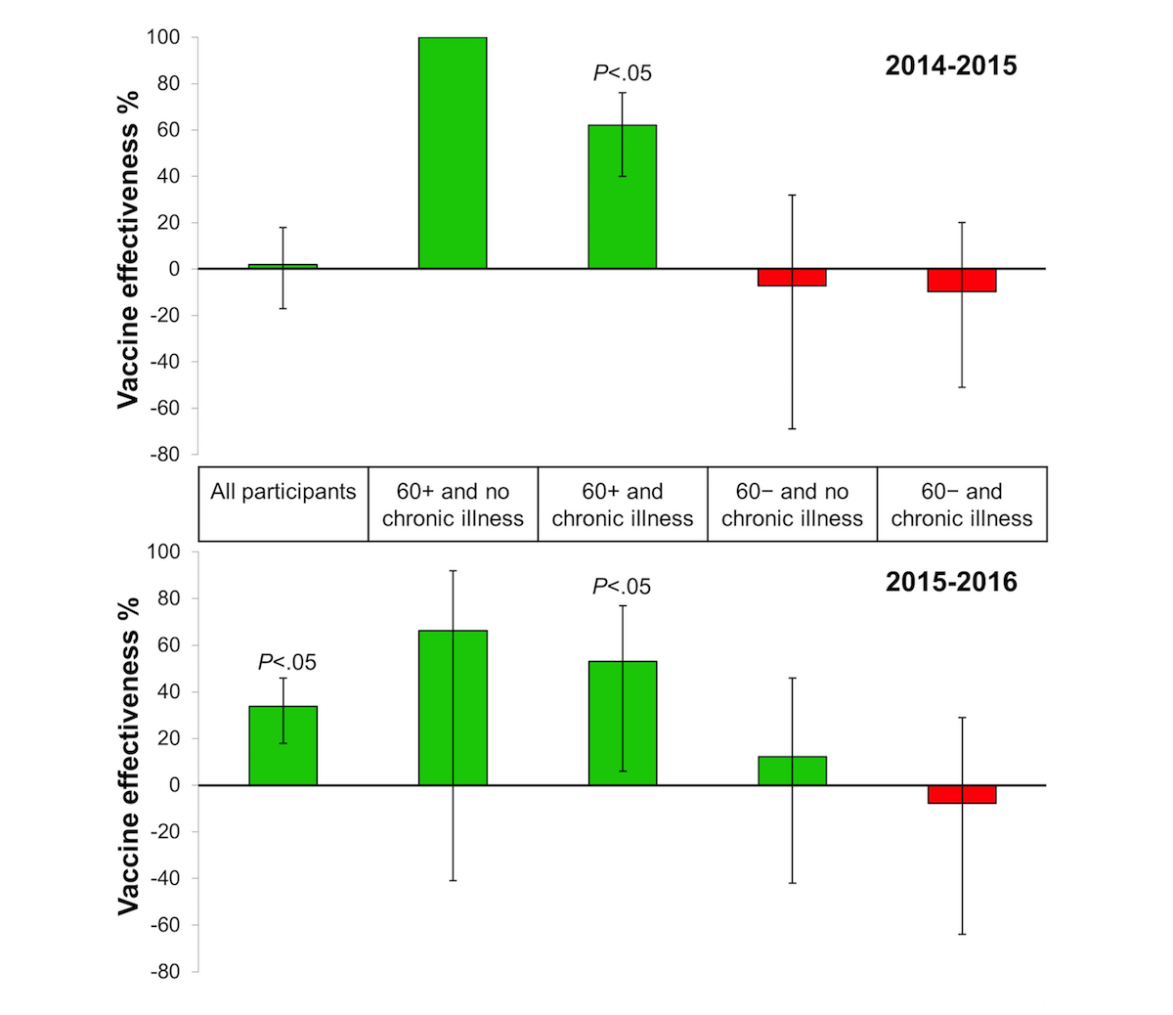
Influenza vaccine effectiveness (VE) in the Netherlands estimated with Influenzanet data during the period of virological confirmed influenza in the 2014-2015 and 2015-2016 seasons. VE(influenza) for all participants and stratified for age and chronic illness, based on self-reported influenza-like illness. The error bars denote the VE 95% CI. (In the "60+ and no chronic illness” stratification it was not possible to calculate the CI in 2014-2015 due to zero ILI cases in the vaccinated group).

At the request of the National Institute for Health and the Environment in the Netherlands, we estimated VE in the Dutch Influenzanet participants for the 2014-15 [49] and 2015-16 [50] seasons, stratified by age and underlying chronic disease, as the number of samples collected by the GPs sentinel networks was too low to allow for these stratified analyses. With 14,000 participants overall yearly, the Dutch Influenzanet database covers 0.08% of the 17 million population in the Netherlands. This large size warrants estimates of VE stratified for particular risk groups, namely, the presence of chronic conditions and older age. Here, we present these estimates, addressing the top two abovementioned considerations. Notably, we considered only the ILI cases in the weeks when there was virological confirmation of influenza by the Dutch Sentinel Practice Network, and of these cases, we used only the number of ILI cases above a seasonal baseline incidence for nonepidemic ILI. By excluding the weeks when there was no virological confirmation of influenza, we excluded the period when most ILI cases were likely because of noninfluenza infections; and by considering only the number of ILI cases above the typical number of ILI cases measured in the absence of circulating viruses, one can therefore obtain a more accurate proxy of VE against influenza. The VE calculated in this way is here designated as VE(influenza), also shown in Figure 3 (see Multimedia Appendix 2 for further details).

If considering the full period of 25 weeks during which Influenzanet collected data (mid-November to the end of April), then the estimated VE against ILI (ie, VE(ILI) in Multimedia Appendix 2) is considerably low. However, VE increases substantially when considering only ILI onsets during the weeks of virological confirmed influenza (17 weeks in 2014-15, 11 weeks in 2015-16) and subtracting the seasonal baseline (ie, VE(influenza) in Table A1 and Figure 3). Namely, the estimated VE in chronic patients almost doubled when considering only the influenza epidemic period with the baseline subtracted, that is, VE(Influenza)~41% in both seasons, compared with when considering the whole data collection period, that is, VE(ILI)~25% in both seasons; even greater VE increases were found for the participants with a chronic condition over 60 years of age (VE=62% [95% CI 40-76] in 2014-15 and 53% [95% CI 6-77] in 2015-16).

The 2014-15 season had a circulating influenza A(H3N2) virus that mismatched with the strain in the vaccine [49]. The 2015-16 season had 2 circulating strains, A(H1N1) and A(H3N2), which appeared to be covered by the vaccine, although there was some mismatch between cocirculating influenza B Victoria compared with B Yamagata in the vaccine [50]. Hence, one would expect not very high VE against influenza in both seasons but higher in 2015-16, given that this season had a better match between the circulating and the vaccine strains. Our results do indeed reflect this pattern, with the estimated VE(influenza) for all participants being greater in 2015-16 (34%; 95% CI 18-46) than in 2014-15 (2%; 95% CI −17 to 18). Interestingly, however, the VE(influenza) in chronic patients was the same in both seasons (41% [95% CI 16-59] in 2015-16 and 41% [95% CI 24-65] in 2014-15). Although in both seasons among chronic patients, the VE(influenza) in participants ≥60 years seemed to be higher than in younger ones (which would seem to go against the current hypothesis that immunosenescense in the elderly results in lower VE [51]), we cannot make direct comparisons with participants aged under 60 years because of the nonstatistically significant estimated VE *P* values. A larger number of participants would be required to make stronger conclusions.

Care should be taken that results of this and the other abovementioned VE studies [22,40,45-47] should not be compared directly, as ILI definitions varied and different methods were used in computing VE.

## Discussion

### Further Studies

The Influenzanet system gathers a variety of valuable data on ILI activity. The analyses of Influenzanet data summarized in this paper reflect only a portion of what is possible. Influenzanet has the potential to monitor the geographical spread of ILI using the postal codes of the participants. Additionally, demographic data could be used to monitor ILI activity in different more or less vulnerable subgroups of the population. Moreover, extra questions can be included in the Web-based intake questionnaire at any time and entire new questionnaires added in any particular season. For instance, a stress-related questionnaire was added in the Netherlands in the 2004-05 season, revealing significant trends between stress/personality and self-reported ILI [52]. Multivariable logistic regression analysis on ILI was performed to test the predictive power of stress and personality. Negative affectivity (Odds ratio [OR] 1.05, *P*=.009), social inhibition (OR 0.97, *P*=.01), and perceived stress (OR 1.03, *P*=.048) predicted ILI reporting. Older age was associated with less ILI reporting (OR 0.98, *P*=.01).

Also in the Netherlands, additional information was collected during the 2009-10 season, on the occurrence of adverse events after administration of the seasonal and pandemic influenza vaccines using either a traditional paper-based survey (for which participants were recruited via GPs) or a Web-based survey (for which participants were recruited via the Dutch Influenzanet; [53]). No significant differences were found in reporting local reactions (OR 0.98, 95% CI 0.88-1.10) or systemic adverse events (OR 1.12, 95% CI 0.99-1.27). There were, however, important differences in the age groups that responded. Namely, the elderly were more represented in the paper-based than in the Web-based survey. Additionally, in both surveys, females reported more local reactions and systemic adverse events than males, the risk of side effects decreased with age, and the presence of a comorbidity increased the risk of local reactions and systemic adverse events.

In other studies of the Influenzanet data in the United Kingdom [54] and the Netherlands [55,56], the analyses of questionnaires related to contact behavior have shown that the changes in contact patterns can explain alterations in disease incidence [54] and that Web-based respondent-driven detection could enhance identification of symptomatic patients by making use of individuals’ local social networks [55,56]. Respondent-driven detection could enable a greater diversity in the age and social status of the participatory surveillance participants, thereby improving the representativeness of the study population and possibly also allow more accurate estimates of the effect of influenza vaccination. One would, however, also need to take into consideration that the proportion of ILI in the study sample could increase because of the participation of a select group of participatory surveillance volunteers with ILI symptoms, as has been observed in the study that tested this approach [56].

Finally, data validation is key to greater acceptance and credibility in the field of public health. In winter, the sentinel GPs that integrate the EISN are asked to take nose and/or throat swabs from a subset of patients with ILI for virological determination. These data inform the national and international decisions by health policy makers. The GP samples, however, do not cover the large part of the population that does not seek medical health care for ILI. A system of self-sampling (ie, swabbing nostrils and/or throat and then sending the swabs for virological testing) among Influenzanet participants could help to overcome this limitation.

This approach has been piloted outside the Influenzanet context by the national public health agencies in both the United Kingdom [57] and Sweden [58]. In the United Kingdom study, a group of 294 callers to the national telephone health helpline (National Health Service Direct) who mentioned colds or influenza were sent a self-sampling kit. They were asked to swab both nostrils and then send the swabs for influenza virus testing. About half the callers sent back the samples, and most did not experience problems in taking the test. The average time between the call and the results of the laboratory was 7 days. The overall influenza-positive rate (16%, 23/142) was lower than in the national virological surveillance system of the United Kingdom (26%), but peak positivity for both the schemes occurred during the same week. This study showed that people can self-sample in a reasonable time frame and that these samples were viable for antigenic characterization and molecular detection, which decreases the need for medical personnel to obtain samples. Self-sampling by the callers provided among the earliest reports of influenza circulating in the community and led to the detection of several strains of the virus [57]. These encouraging results have led to a self-sampling study with Influenzanet participants being planned in the United Kingdom to strengthen the validation of the participatory surveillance data.

We also plan to integrate the Influenzanet data with social networks, news streams, health forums, clinical records, and routine data to have a better understanding of the socioeconomic aspects of ILI epidemics in Europe and some behavioral insights on, for example, attitude toward influenza vaccination.

### Early Warning

Detecting an earlier rise of ILI activity in certain subgroups could make Influenzanet a fast early-warning system. Indeed, it is often suggested that self-reporting surveillance systems might be able to detect changes in disease activity earlier than the traditional surveillance systems [32,33,59,60]. This is because of the self-reported data becoming instantaneously available for automated analyses, whereas in the traditional systems there is a delay from when data are collected in the medical facilities until they are available for centralized analyses. However, run-time detection of disease activity above baseline should not be confused with detection of a newly emerging disease. For a participatory surveillance system such as Influenzanet to become a viable system for early warning of the first cases of a new disease, a greater proportion of the population needs to be engaged [32]; so far the platform has been able to recruit at the most 0.08% of a country's population. For Influenzanet to become a European-level early warning system for serious cross-border health threats, all European countries would need to participate. Additional research is also needed to identify the best way to differentiate a signal caused by an influenza epidemic with one caused by a “new disease” of a respiratory nature, or producing respiratory symptoms, and how to set that threshold.

### Continuity and Sustainability

#### Recruitment and Participation

The added value that the Influenzanet system brings to ILI surveillance depends on recruiting and retaining as many active participants as possible, covering a wide geographical area and from diverse age and risk groups.

Although specific Influenzanet recruitment strategies vary between countries, they tend to be based on mass communication [61,62]. Participants are recruited through press releases, direct mailings to schools, and interviews on national and local television and radio, in national and regional newspapers, and on social media. Schools are also provided with educational material on influenza to promote incorporation of disease surveillance concepts in science classes. At the beginning of each season, all participants from previous seasons are sent an email, inviting them to participate again by completing an intake questionnaire for the new season.

An important cornerstone for enhancing active participation is the information feedback offered to participants. The participants receive weekly emails containing a newsletter with country-specific data and influenza-related news articles written by professional science journalists, which helps keeping them involved and motivated.

From a science communication point of view, an interesting finding from the Dutch project is that Influenzanet has been able to attract and keep engaged many people without previous experience in scientific research, citizen science projects, or science activities in their daily lives. This contradicts previous findings that participants may be restricted to a self-selected group with previous experience and interest in science [63]. One way the Dutch Influenzanet reaches out to both current and potential participants is by having a well-known science communicator serve as the ambassador of the project. Other Influenzanet countries have also experienced the importance of having science communication experts working alongside scientists in the project to reach the general public. It is important to emphasize how participants have contributed to the findings and the success of the project and that their continued contributions are valued [4].

#### Sustainability

Influenzanet is low-cost to run in comparison with traditional systems. However, it is not free of costs and needs active support to continue, especially where self-swabbing, in addition to self-reporting, is concerned. Funding for Influenzanet is also vital for the project's maintenance, in particular to keep participants actively engaged via the weekly newsletter and social media interaction, to further improve the national platforms and to keep recruiting new participants by providing the media with the latest news and results. Also, acceptance must be sought among influenza health care professionals so that the results can be displayed and used along with other surveillance and response systems.

From 2009 to 2013, the European Union’s FP7 project, EPIWORK, made it possible to extend the Influenzanet system, which at the time included only the Netherlands, Belgium, Portugal, and Italy, into 6 additional European countries. Influenza monitoring with self-reporting volunteers is now active in 10 European countries [39]. With the exception of the Netherlands and Belgium, where Influenzanet is run by a small private company, in all other countries it is coordinated by teams in national research and/or public health institutions.

### Conclusions

Influenzanet can complement traditional health care–based systems by providing data that are not otherwise available. Influezanet is able to achieve this because it allows collecting data also from people who do not seek health care (and are therefore not accounted in traditional ILI surveillance), and it additionally gathers detailed information about the participants that is not routinely collected elsewhere. Due to its uniform nature across countries, it both allows for direct comparisons of ILI activity between countries and provides a platform to monitor the geographical spread of ILI throughout Europe. Moreover, Influenzanet provides an important channel for influenza awareness and health literacy in Europe. With its speed and flexibility, the system could be extended to detect diseases other than influenza, including those that emerge in low-income settings such as dengue, leptospirosis, severe acute respiratory syndrome, Ebola, Middle East respiratory syndrome, and Zika virus, and where community engagement is vital [35,64-66]. If so, this novel Internet monitoring system based on voluntary participants could develop into an important weapon to fight influenza as well as other contagious diseases globally. Influenzanet in Europe is an example of best practice here, not only by engaging citizens to report information that enables to complement the data obtained by traditional disease surveillance systems but also by providing a flexible and wide reach health literacy channel, delivering back reliable and updated information to the population about disease activity, transmission, and prevention strategies. During the second International Workshop on Participatory Surveillance (Amsterdam, the Netherlands, April 15-17, 2013) a letter of intent on cooperation and data exchange has been agreed between Influenzanet, Flu Tracking, and Flu Near You. The aim is to achieve a worldwide “disease radar,” whereby everyone is invited to fill in their own health status.

## Appendix

Multimedia Appendix 1 Influenzanet intake and weekly symptoms' questionnaires used since 2012-13 (UK version 121101).

Multimedia Appendix 2 Influenza vaccine effectiveness analyses for the Netherlands 2014-15 and 2015-16.

## Abbreviations

ECDC: European Centre for Disease Control and Prevention
EISN: European Influenza Surveillance Network
GP: general practitioner
ILI: influenza-like illness
I-MOVE: Influenza-Monitoring Vaccine Effectiveness
LINH: Landelijk Informatie Netwerk Huisartsenzorg
OR: odds ratio
SD: standard deviation
TESSy: The European Surveillance System
VE: vaccine effectiveness
WHO: World Health Organization
