# Supplementary material for: Influenzanet: Citizens Among 10 Countries Collaborating to Monitor Influenza in Europe
Source: JMIR Public Health Surveill. 2017 Sep 19;3(3):e66. doi: 10.2196/publichealth.7429 (PMC5627046; doi:10.2196/publichealth.7429)
Supplement: Multimedia Appendix 1 [file publichealth_v3i3e66_app1.pdf]

Influenzanet

Influenzanet Agreed Questionnaire 2012

In blue: comments regarding technical implementation and national application.

In red: notes with information on the question for the participants.

Where the options is “pick only one” (e.g. Intake Q1) = *filled* bullet points;
Where it is “pick all that apply” (e.g. Intake Q5) = *open* bullet points.

This latter option will need some built in checks, since “No” is often an option we would want to retain.

General questionnaire

Individuals who have ***un****subscribed* from the newsletter/project or stopped reporting (no report for at least 6 weeks), should be prompted to fill in this short, separate questionnaire.

I have stopped participating because:

- I have not had any symptoms to report
- I got out of the habit
- I do not have time
- The weekly questionnaire takes too long
- The newsletter is not interesting
- The website is not interesting
- I am tired of the study
- I have been away/out of town
- I have not had internet access
- I have been experiencing technical difficulties
- I am almost never sick
- I do not think flusurvey is the right way to track influenza
- I am tired of hearing about influenza
- Other reason

Why are we asking this?
This information will help us improve the survey in the future.

Intake questionnaire

Intake Q0

Question to be asked ***when*** adding another participant. For *each* additional person that a person registered this question should be asked.

If a person just registers and enters data for himself, then this question should *not* be asked.

Is this person a member of your household?

- Yes
- No

If you are filling in the survey on behalf of someone else, then make sure that you have the consent of that person to do so

Why are we asking this?
Flu is thought to spread very effectively at home, so it's important to know which *Flusurvey* (or *Influweb* or *Influensakoll* or…) participants live in the same household.

How should I answer it?
A household member is defined as a someone (not necessarily related) who lives at the same address as you and who shares cooking facilities and a living room, sitting room or dining area.

Intake Q1

What is your gender?

- Male
- Female

Why are we asking this?
To find out whether the chance of getting flu is different between men and women.

Intake Q2

What is your date of birth (month and year)?

Two drop-down lists

Why are we asking this?
The chance of getting flu and the risk of more serious complications vary by age.

Intake Q3

Should be pre-filled for multiple users of a *single* account, if they're in the same household.

This is the NL/UK version. For countries which have permission to collect the *whole* post-code then this should be re-worded, and the data collected to allow.

What is the first part of your home postal code?

XXXX / the whole post-code to be collected

Why are we asking this?
To check how representative our sample is, and to see whether the chance of getting of flu varies across the country.

How should I answer it?
Please choose the first part of the post-code (the part before the space).

Intake Q4

What is your main activity?

- Paid employment, full-time
- Paid employment, part-time
- Self-employed (businessman, farmer, tradesman, etc)
- Attending daycare/school/college/university
- Home-maker (e.g. housewife)
- Unemployed
- Long-term sick-leave or parental leave
- Retired
- Other

Why are we asking this?
To check how representative our sample is compared to the population as a whole, and to find out whether the chance of getting flu is different for people in different types of occupation.

How should I answer it?
Please tick the box that *most closely* resembles your main occupation. For pre-school children who don't go to daycare tick the "other" box.

Intake Q4b

If "Yes, Paid employment full time", or "Yes, paid employment part time" to Q4, or "Self-employed (businessman, farmer, tradesman, etc), or "Attending daycare/school/college/university"

What is the first part of your school/college/workplace postal code (where you spend the majority of your working/studying time)?

- XXXX
- I don't know/can't remember
- Not applicable (e.g. don't have a fixed workplace)

Why are we asking this?
To find out roughly how far you travel on a regular basis.

How should I answer it?
Please choose the *first* part of the post-code (the part before the space).

Intake Q4c

If "Yes, Paid employment full time", or "Yes, paid employment part time" to Q4, or "Self-employed (businessman, farmer, tradesman, etc).

Trigger free text if "other" checked, allow the users to write in their occupation

Which of the following descriptions most closely matches with your main occupation?

- Professional (e.g. manager, doctor, teacher, nurse, engineer)
- Office work (e.g. admin, finance assistant, receptionist, etc)
- Retail, sales, catering and hospitality and leisure (e.g. shop assistant, waiter, bar-staff, gym instructor etc)
- Skilled manual worker (e.g. mechanic, electrician, technician)
- Other manual work (e.g. cleaning, security, driver)
- Other

Why are we asking this?
To check how representative our sample is compared to the population as a whole and to find out whether the chance of getting flu is different for people in different types of occupation.

How should I answer it?
Please tick the box that *most closely* resembles your main occupation.

Intake Q4d (to be asked for those aged 16 and over)

This is the UK version. The 2nd bullet point is exams taken at the time of early school leaving (16 in the UK), the 3rd bullet point for late school leaving (18 in the UK). The others are for degrees, or higher qualifications, which should be fairly comparable across Europe.<br><br>The questions that you should ask at the bullets 2 and 3, are related to exams taken at early and late school leaving ages.

What is the highest level of formal education qualification that you have?

If you are still in education, then please tick this box with the appropriate highest level that you have already achieved**.**

- I have no formal qualifications
- GCSE's, O'levels, CSEs or equivalent
- A-Levels or equivalent (e.g. Highers, NVQ Level3, BTEC)
- Batchelors Degree (BA, BSc) or equivalent (e.g. HND, NVQ Level 4)
- Higher Degree or equivalent (e.g. Masters Degree, PGCE, PhD, Medical Doctorate, Advanced Professional Awards)
- I am still in education

Why are we asking this?
To check how representative our sample is compared to the population of the UK (Italy, Belgium, etc….) as a whole.

How should I answer it?
Please choose the box that represents your **HIGHEST** level of educational achievement. The different options roughly equate to:

- No qualifications
- school-leaving exams at around 16 years of age
- school-leaving exams at around 18 years of age
- University degree, or equivalent professional qualification
- Higher degree or advanced professional qualification

If you are an adult who is currently undergoing **part-time training** (e.g. night-school) then tick the box that represents your **current highest level of education**.

Intake Q5

Needs to have a check so that participants can't give inconsistent answers

Except people you meet on public transports, do you have contact with any of the following during the course of a typical day?

(Select all options that apply, if any)

- More than 10 children or teenagers over the course of the day

- More than 10 people aged over 65 over the course of day
- Patients
- Groups of people (more than 10 individuals at any one time)
- None of the above

Why are we asking this?

To find out whether you are likely to be exposed to more flu than the average person (e.g. work with children, or patients)

How should I answer it?

Groups of people could include any setting where you come into contact with large numbers of people at once, e.g. a teacher who may contact many children in a day

Intake Q6

This should be a table with the 1st column being the age groups (as above), and the 2nd column being numbers, 0,1,2,3,4,5+. It should be **PRE-FILLED with zeros**.

Preferably to be pre-filled for multiple users of a single account, if they're in the same household?

INCLUDING YOU, how many people in each of the following age groups live in your household?

Drop down menus for each of:

0-4 years

5-18 years

19-44 years

45-64 years

65+ years

Why are we asking this?

Members of larger households, or those with children, may be more likely to catch flu than others.

How should I answer it?

A household is defined as a group of people (not necessarily related) living at the same address who share cooking facilities and share a living room, sitting room or dining area.

Intake Q6b (If any in household are aged 0-18, including participant):

Ideally, this question would only be asked if they have children aged 0-18 – or if they're aged 0-18 themselves, and the answer would be a drop-down menu with the right number of options.

Preferably, this be pre-filled for multiple users of a single account, if they're in the same household

How many of the children in your household go to school or day-care?

Drop-down menu

Why are we asking this?

Attending school or day-care may be a risk for acquiring flu and similar illnesses. We would like to check this.

How should I answer it?

If your child attends regular school or day-care (even if this is just one day a week) then please answer yes to this. Attendance of clubs and activities does not count – even if regular.

Intake Q7

What is your *main* means of transportation?

- Walking
- Bike
- Motorbike/scooter
- Car
- Public transportation (bus, train, tube, etc)
- Other

Why are we asking this?

It has been suggested that using public transport may be a risk for flu. We would like to check this.

How should I answer it?

Tick the option that best represents your *most* normal mode of transport.

Intake Q7b

On a normal day, how much time do you spend on public transport (bus, train, tube, etc)?

- No time at all
- 0-30 minutes
- 30 minutes – 1.5 hours
- 1.5 hours – 4 hours

- Over 4 hours

Why are we asking this?

It has been suggested that using public transport may be a risk for getting flu. We would like to check this.

How should I answer it?

Think of a typical day. If you use several different forms of public transport each day, remember to include all journeys. Don't include taxis or other forms of private transport.

Intake Q8 (not a core question)

How often do you have common colds or flu-like diseases?

- Never
- Once or twice a year
- Between 3 and 5 times a year
- Between 6 and 10 times a year
- More than 10 times a year
- I don't know

Intake Q9

Note that the years will need to be changed each year.

Did you receive a flu vaccine during the last autumn/winter season (2011-2012)?

- Yes
- No
- I don't know/can't remember

Why are we asking this?

We would like to be able to work out how much protection the vaccine gives. We would also like to find out if there is some protection from vaccines received in previous years.

How should I answer it?

Answer yes if you received the vaccine last year (during the autumn/winter of 2011/2012

Intake Q10

Note that the years will need to be changed each year.

Have you received a flu vaccine this autumn/winter season (2012-2013)?

- Yes [go to questions Q10b and Q10c]
- No [go to question Q10d]
- I don't know/can't remember

Why are we asking this?

We would like to be able to work out how much protection the vaccine gives.

How should I answer it?

Report yes, if you received the vaccine this season, usually in the autumn. If you get vaccinated after filling in this questionnaire, please return to this and update your answer.

Intake Q10b: (if "Yes" to Intake Q10, follow-up question)

Note that the years will need to be changed each year.

When were you vaccinated against flu this season (2012-2013)?

- XX/XX/XXXX
- I don't know/can't remember

Why are we asking this?

Knowing when people are vaccinated tells us how well the vaccination programme is being carried out.

How should I answer it?

Please try and answer as accurately as possible. If you do not know the precise date, please give your best estimate. For instance, you might remember the month, then try and remember if it was at the beginning or the end of the month. Were there any significant events (e.g. a holiday or a birthday) that might help jog your memory?

Intake Q10c: (if "Yes" to Intake Q10, follow-up question)

Note that risk groups (option 1) are UK-specific and may need adjusting for other countries.

What were your reasons for getting a seasonal influenza vaccination this year?

(Select all options that apply)

- I belong to a risk group (e.g. pregnant, over 65, underlying health condition, etc)
- Vaccination decreases my risk of getting influenza
- Vaccination decreases the risk of spreading influenza to others

- My doctor recommended it
- It was recommended in my workplace/school
- The vaccine was readily available and vaccine administration was convenient
- The vaccine was free (no cost)
- I don’t want to miss work/school
- I always get the vaccine
- Other reason(s)

Intake Q10d: (if “No” to Intake Q10, follow-up question)

What were your reasons for NOT getting a seasonal influenza vaccination this year?

(Select all options that apply)

- I am planning to be vaccinated, but haven’t been yet
- I haven’t been offered the vaccine
- I don't belong to a risk group
- It is better to build your own natural immunity against influenza
- I doubt that the influenza vaccine is effective
- Influenza is a minor illness
- I don’t think that I am likely to get influenza
- I believe that influenza vaccine can cause influenza
- I am worried that the vaccine is not safe or will cause illness or other adverse events
- I don’t like having vaccinations
- The vaccine is not readily available to me
- The vaccine is not free of charge
- No particular reason
- Although my doctor recommended a vaccine, I did not get one
- Other reason(s)

Why are we asking this?

We would like to know why some people get vaccinated and others do not

How should I answer it?

Tick all those reasons that were important in your decision.

Intake Q11

Needs to have a check so that participants can't give inconsistent answers

Do you take regular medication for any of the following medical conditions?
(Select all options that apply)

- No
- Asthma
- Diabetes
- Chronic lung disorder besides asthma e.g. COPD, emphysema, or other disorders that affect your breathing
- Heart disorder
- Kidney disorder
- An immunocompromising condition from treatment or illness including splenectomy, organ transplant, acquired immune deficiency, cancer treatment

Why are we asking this?

This question allows us to find out whether you have other medical conditions that may increase your risk of having more severe illness if you are infected with flu.

How should I answer it?

Only answer "yes" if you take regular medication for your medical problem. If, for instance, you only occasionally take an asthma inhaler, then do not answer "yes" for asthma.

Intake Q12

Only asked of women between ages 15 and 50)

Are you currently pregnant?

- Yes [go to question Q12]
- No
- Don't know/would rather not answer

Why are we asking this?

Pregnancy can result in more severe illness if you are infected with flu.

How should I answer it?

Answer yes if you are pregnant today.

Intake Q12b: (if "Yes" to Intake Q12, follow-up question)

Which trimester of the pregnancy are you in?

- First trimester (week 1-12)
- Second trimester (week 13-28)
- Third trimester (week 29-delivery)
- Don't know/would rather not answer

Why are we asking this?

The stage of pregnancy *might* alter your risk of severe flu if you are infected, although this is not very clear.

How should I answer it?

Which trimester are you in today?

Intake Q13

Do you smoke tobacco?

- No
- Yes, occasionally
- Yes, daily, fewer than 10 times a day
- Yes, daily, 10 or more times a day
- Don't know/would rather not answer

Why are we asking this?

Smoking might make you more likely to get a more severe dose of flu. We would like to test this.

How should I answer it?

Please answer as accurately as possible. If you smoke other products (e.g. a pipe or cigars), then indicate roughly how many times a day.

Intake Q14

Needs to have a check so that participants can't give inconsistent answers

Do you have one of the following allergies that can cause respiratory symptoms?

(Select all options that apply)

- Hay fever
- Allergy against house dust mite
- Allergy against domestic animals or pets

- Other allergies that cause respiratory symptoms (e.g. sneezing, runny eyes)
- I do not have an allergy that causes respiratory symptoms

Why are we asking this?

Some allergic reactions can have similar symptoms to respiratory infection.

How should I answer it?

Tick all the options that apply. We are only interested in those allergies that cause respiratory symptoms (i.e. sneezing, runny nose, runny eyes).

Intake Q15 (not a core question)

Needs to have a check so that participants can’t give inconsistent answers

Do you follow a special diet?

(Select all options that apply)

- No special diet
- Vegetarian
- Veganism
- Low-calorie
- Other

Intake Q16 (not a core question)

Needs to have a check so that participants can’t give inconsistent answers

Do you have pets at home?

(Select all options that apply)

- No
- Yes, one or more dogs
- Yes, one or more cats
- Yes, one or more birds
- Yes, one or more other animals

Symptoms questionnaire

If you are filling this in on behalf of someone else, please answer all the questions as if you are that person.

Weekly Q1

- Needs to have a check so that participants can't give inconsistent answers
- **If report any symptoms, check database**:
  was the participant STILL ILL when they last completed the Weekly survey (i.e. they said "I am still ill" in response to Weekly Q4 below last time?) **AND** have fewer than 15 days elapsed since they last completed the Weekly Survey **AND** fewer than 15 days have elapsed since their previous date of symptom onset
- If **Yes**: continue with Weekly Q2
- If **No** or **Don't know**: then treat this as a "new" bout of illness, continue with Weekly Q3
- Of course, this only applies to people who have previously participated

Have you had any of the following symptoms since your last visit (or in the past week, if this is your first visit)?

(Select all options that apply)

- No symptoms
- Fever
- Chills
- Runny or blocked nose
- Sneezing
- Sore throat
- Cough
- Shortness of breath
- Headache
- Muscle/joint pain
- Chest pain
- Feeling tired or exhausted (malaise)
- Loss of appetite
- Coloured sputum/phlegm
- Watery, bloodshot eyes
- Nausea
- Vomiting
- Diarrhoea
- Stomach ache
- Other

Why are we asking this?

The key part of this survey is tracking people's symptoms.

How should I answer it?

For people with chronic (long-term) illnesses, only tick changes in symptoms. So, if you have chronic shortness of breath (for instance) then only tick this box if this has got worse recently.

Weekly Q2 (If the participant was STILL ILL on their last visit and has reported symptoms this time):

The "don't know" option here might not apply to people filling it in for themselves but is relevant for people managing accounts for other people.

If **NO** or **DON'T KNOW:**
This means that the current symptoms are the start of a "new" illness: continue with Weekly Q3

If **YES:**
This means that the current symptoms are the continuation of the same bout of illness as the previous visit. The remainder of the symptoms questionnaire (onset date, further details about symptoms, details about seeking medical attention, treatment, time off work/school) can be *pre-filled* with their previous answers.

The point of the pre-filling is to make the survey quicker and easier for participants who have longer-duration symptoms.

"On DATE OF LAST VISIT you reported that you were still ill with symptoms that began on DATE OF FIRST SYMPTOMS REPORTED PREVIOUSLY.

Are the symptoms you reported today part of the same bout of illness?

- Yes
- No
- I don't know/can't remember

Why are we asking this?

To make filling out the rest of the survey quicker for you.

How should I answer it?

If you believe that the symptoms you have reported today are caused by the same bout of illness as your previous symptoms, please tick "yes".

To save you time, we have filled in the information you gave us previously about your illness. Please check that it is still correct, and make any changes – for instance, if you have visited a doctor or taken additional time off work since you last completed the survey.

Weekly Q3 (if symptoms)

When did the first symptoms appear?

- Choose date XX/XX/XXXX
- I don't know/can't remember

Why are we asking this?

To help us work out the number of cases of flu that arise each day.

How should I answer it?

Please give as accurate an estimate as possible.

Weekly Q4 (if symptoms)

When did your symptoms end?

- Choose date XX/XX/XXXX
- I don't know/can't remember
- I am still ill

**Why are we asking this?**<br>Using the beginning and end dates of symptoms we can work out how long respiratory infections last.<br>**How should I answer it?**<br>Please give as accurate an estimate as possible.

Weekly Q5 (if symptoms)

Did your symptoms develop suddenly over a few hours?

- Yes
- No
- I don't know/can't remember

**Why are we asking this?**<br>Sudden onset of symptoms is believed to be common for flu.<br>**How should I answer it?**<br>Tick yes if your symptoms appeared over a few hours rather than gradually developing over a few days.

Weekly Q6 (if fever)

When did your fever begin?

- Choose date XX/XX/XXXX
- I don't know/can't remember

**Why are we asking this?**<br>Fever is very important for diagnosing flu, so we want to know when this started.<br>**How should I answer it?**<br>Please give as accurate an estimate as possible.

WEEKLY Q6b (extra non-core question) (if fever)

Did your fever develop suddenly over a few hours?

- Yes
- No
- Don't know

Why are we asking this?

Flu is often associated with a sudden onset of fever

How should I answer it?

Tick yes if your fever appeared over a few hours rather than gradually developing over a few days.

Weekly Q6c (if symptoms)

Did you take your temperature?

- Yes [go to Weekly Q6c]
- No
- I don’t know

Why are we asking this?

Flu often causes a high temperature. However, not everyone takes their temperature when they are ill.

How should I answer it?

Answer yes, if you took your temperature using a thermometer.

Weekly Q6d(if symptoms) and (if took temperature): follow-up question

What was your highest temperature measured?

- Below 37° C
- 37° - 37.4°C
- 37.5° - 37.9°C
- 38° – 38.9°C
- 39° - 39.9°C
- 40°C or more

- I don’t know/can’t remember

**Why are we asking this?**<br><br>Flu often causes a high temperature.<br><br>**How should I answer it?**<br><br>Give the highest temperature you recorded during this episode of illness.

Weekly Q7 (if symptoms)

Will need to be adjusted to suit different countries.<br><br>Needs to have a check so that participants can’t give *in*consistent answers

Because of your symptoms, did you VISIT (see face to face) any of medical services?

(Select all options that apply)

- No
- GP or GP’s practice nurse
- Hospital admission
- Hospital accident & emergency department/out of hours service
- Other medical services
- No, but I have an appointment scheduled

**Why are we asking this?**<br><br>To find out whether people contact the health services because of their symptoms.<br><br>**How should I answer it?**<br><br>Tick all of those that apply. If you are due to see attend, then tick the final option.

Weekly Q7b (if symptoms)

This should be asked for each option ticked in the previous question (not including “No” or “Not yet”)<br><br>Table for each entry in the previous question. And then drop-down within each cell for the delays. Last year we had it as a table of radio boxes, but it displayed the same table no matter which options were chosen in Weekly Q7 – it would be nice if it only displayed the options selected in Weekly Q7

How soon after your symptoms appeared did you first visit this medical service?

- Same day

- 1 day
- 2 days
- 3 days
- 4 days
- 5-7 days
- More than 7 days
- I don't know/can't remember

Why are we asking this?

To find out how quickly people with symptoms are seen by the health services.

How should I answer it?

Only record the time until your FIRST contact with the health services.

Weekly Q8 (if symptoms)

This is the UK version. Will need to be adjusted to suit different countries.

Needs to have a check so that participants can't give inconsistent answers

Because of your symptoms, did you contact via TELEPHONE or INTERNET any of the following?

(Select all options that apply)

- No
- GP – spoke to receptionist only
- GP – spoke to doctor or nurse
- NHS Direct / NHS 24 / NHS Choices
- NPFS
- Other

Why are we asking this?

To find out whether people contact the health services because of their symptoms.

How should I answer it?

Tick all options that apply

Weekly Q8b (if symptoms)

This should be asked for each option ticked in the previous question (not including "No")<br><br>Preferably, it only displays the options selected in Weekly Q8.

How soon after your symptoms appeared did you first contact via telephone or internet any of the following?

- Same day
- 1 day
- 2 days
- 3 days
- 4 days
- 5-7 days
- More than 7 days
- I don't know/can't remember

**Why are we asking this?**<br><br>To find out how quickly people with symptoms contact the health services.<br><br>**How should I answer it?**<br><br>Only record the time until your FIRST contact with the health services.

Weekly Q9 (if symptoms)

This is adapted to the UK situation. Other countries will need to adapt the brand names of the commonly used pain killers or antipyretics<br><br>Needs to have a check so that participants can't give inconsistent answers.

Did you take medication for these symptoms?

(Select all options that apply)

- No medication
- Pain killers (e.g. paracetamol, lemsip, ibuprofen, aspirin, calpol, etc)
- Cough medication (e.g. expectorants)
- Antivirals (Tamiflu, Relenza)
- Antibiotics
- Other
- Extra country specific options – mapped to "other" in standardised database

- I don't know/can't remember

Why are we asking this?

To find out who gets treated, and how effective treatment is.

How should I answer it?

Only record those medications that you used because of this bout of illness. If you are on other medications because of a pre-existing illness then do not record these.

Weekly Q9b (if antivirals were taken): follow-up question

How long after the beginning of your symptoms did you start taking antiviral medication?

- Same day (within 24 hours)
- 1 day later
- 2 days later
- 3 days later
- 4 days later
- 5-7 days later
- More than 7 days later
- I don't know/can't remember

Why are we asking this?

Antivirals are thought to be most effective if taken quickly after disease onset.

How should I answer it?

Report the time until you first started taking antivirals (which may not be the same day as you got your prescription).

Weekly Q10 (if symptoms)

Did you change your daily routine because of your illness?

- No
- Yes, but I did not take time off work/school
- Yes, I took time off work/school

Why are we asking this?

To measure how people's daily lives are affected by their symptoms.

How should I answer it?

We want to know if you were absent from work or school as a result of your illness, or if you changed your routine in other ways (e.g. stopped attending clubs).

Weekly Q10b (if symptoms) & (if taken time off work/school): follow-up question

Are you still off work/school?

- Yes
- No
- Other (e.g. I wouldn't usually be at work/school today anyway)

Why are we asking this?

To estimate the average amount of time that people take off work, we need to know if people are still off work.

How should I answer it?

Tick "yes" if you would be at work/school today if you were not currently ill.

Weekly Q10c (if symptoms) & (if taken time off work/school): follow-up question

How have you been off work/school for?

- 1 day
- 2 days
- 3 days
- 4 days
- 5 days
- 6 to 10 days
- 11 to 15 days
- More than 15 days

Why are we asking this?

To measure the effect of symptoms on people's daily lives.

How should I answer it?

Only count the days that you normally would have been in school or work (e.g. don't count weekends).

Weekly Q11 (if symptoms)

What do you think is causing your symptoms?

- Flu or flu-like illness
- Common cold
- Allergy/hay fever
- Asthma
- Gastroenteritis/gastric flu
- Other
- I don't know

Why are we asking this?

To help find out if our assessment of your illness based on your symptoms matches what you believe to be the cause. You might have a better idea of what is causing your illness than our computer algorithms.

How should I answer it?

If you are reasonably sure about what is causing your symptoms, please tick the appropriate box. Otherwise, please tick "I don't know".

Contact questionnaire

Flu is spread via social contacts. Measuring how we meet each other helps understand and predict flu epidemics. Think about the all people you met yesterday and where you met them.

Contact Q1

Each select box has options 0 (pre-filled), 1, 2, 3, 4, 5, 6, 7, 8, 9, 10, 11, 12, 13, 14, 15, 16-24, 25-49, 50-99, 100+

How many people did you have conversational contact with yesterday (talking face to face)?

Table of selects with

- Columns: 0-4 years; 5-18 years; 19-44 years; 45-64 years; 65+ years
- Rows: Home; Work; Other

Why are we asking this?

To find out whether people who have many contacts (perhaps with certain age groups) are more likely to experience symptoms.

How should I answer it?

Only record people in one setting. So if you saw someone at work and socialising, then only record them in the place where you spent *most* time together. “Home” means your home. “Work” means you place(s) of work (or school for younger participants).

Anyone you have had a conversational contact AND a physical contact with should be recorded both here and in the next question – i.e. they should be included twice.

Contact Q2

Each select box has options 0 (pre-filled), 1, 2, 3, 4, 5, 6, 7, 8, 9, 10, 11, 12, 13, 14, 15, 16-24, 25-49, 50-99, 100+

How many people did you have physical contact with yesterday (skin-to-skin contact, e.g. handshake, kiss)?

Table of selects with

- Columns: 0-4 years; 5-18 years; 19-44 years; 45-64 years; 65+ years
- Rows: Home; Work; Other

Why are we asking this?

Illnesses might be spread via touch (e.g. when shaking hands).

How should I answer it?

Only record people in one setting. So if you saw someone at work and socialising, then only record them in the place where you spent *most* time together. “Home” means your home. “Work” means you place(s) of work (or school for younger participants).

Anyone you have had a conversational contact AND a physical contact with should be recorded both here and in the previous question – i.e. they should be included twice.

Contact Q3

How much time did you spend on public transport (e.g. bus, train, underground) yesterday?

- No time at all
- 0-30 minutes
- 30 minutes – 1.5 hours
- 1.5 hours – 4 hours
- Over 4 hours

Why are we asking this?

It has been suggested that using public transport may be a risk for getting flu. We would like to check this.

How should I answer it?

Don’t include taxis, or other forms of private transport.

Contact Q4

How long did you spend in an enclosed indoor space (e.g. office, classroom, bar, cinema) with more than 10 other people yesterday?

(Not including public transport)?

- No time at all
- 0-30 minutes
- 30 minutes – 1.5 hours
- 1.5 hours – 4 hours
- Over 4 hours

Why are we asking this?

Attending busy places is thought to be a risk factor for getting flu .We would like to check this.

How should I answer it?

Remember not to include time spent on public transport, as we ask this in the previous question.

Contact Q5

Version in miles for UK and Ireland

What was the furthest distance from home that you travelled yesterday?

- Under 1 mile
- 1-4 miles
- 5-9 miles
- 10-29 miles
- 30-100 miles
- Over 100 miles

Why are we asking this?

To find out how far you travel, and whether there is a link with catching flu.

How should I answer it?

If you did not leave home, then tick the "under 1 mile" option.

Contact Q5b

Version in kilometres for continental Europe

What was the furthest distance from home that you travelled yesterday?

- Under 1 km
- 1-5 km
- 6-15 km
- 16-50 km
- 51-150 km
- Over 150 km

Why are we asking this?

To find out how far you travel, and whether there is a link with catching flu.

How should I answer it?

If you did not leave home, then tick the "under 1 km" option.
