# Supplementary material for: Influenzanet: Citizens Among 10 Countries Collaborating to Monitor Influenza in Europe
Source: JMIR Public Health Surveill. 2017 Sep 19;3(3):e66. doi: 10.2196/publichealth.7429 (PMC5627046; doi:10.2196/publichealth.7429)
Supplement: Multimedia Appendix 2 [file publichealth_v3i3e66_app2.pdf]

INFLUENZANET: Citizens Among Ten Countries Collaborating to Monitor Influenza in Europe

Carl E Koppeschaar, BSc; Vittoria Colizza, PhD; Caroline Guerrisi, MPH; Clement Turbelin, MD PhD; Jim Duggan, PhD; W John Edmunds, PhD; Charlotte Kjelsø, MSc; Ricardo Mexia, MD MPH; Yamir Moreno, PhD; Sandro Meloni, PhD; Daniela Paolotti, PhD; Daniela Perrotta, MSc; Edward van Straten, BSc; Ana O Franco, DVM, PhD

Multimedia Appendix 2

Influenza vaccine effectiveness analyses for the Netherlands 2014-15 and 2015-16

Here, we present the calculation of vaccine effectiveness (VE) for the Netherlands in 2014-15 and 2015-16 stratified for particular risk groups, namely chronic conditions and older age.

Influenza-like illness was defined as the acute onset of fever (measured temperature ≥38 °C), with muscle pain or headache and cough or sore throat (designated as ILI^{hist} case definition in the **Text box Case Definitions**). The analyses included only the participants that submitted at least 3 symptom surveys throughout the monitoring season, designated as "active" participants.

Weekly ILI incidences of "active" participants were used, causing a slight difference in the mean number of participants for the different periods considered. The weekly ILI incidence was defined as the number of participants who reported the onset of ILI in that week, divided by the person-time of active participation during the same week. The person-time of active participation per week was defined as the number of participants who were active during any day(s) of that week multiplied by the fraction of that week during which each participant was active.

We considered only the ILI cases in the weeks when there was virological confirmation of influenza by the Dutch Sentinel Practice Network, and of these cases, we used only the number of ILI cases above a seasonal baseline incidence for non-epidemic ILI.

The baseline corresponds to the typical number of ILI cases measured in the absence of circulating influenza viruses and was determined as *baseline = 275 + 75 cosine ((week number - 2) / 52) x 360)*. These values were chosen as they gave the best fit for the Netherlands results in 13 seasons (also considering ILI cases in summer when the Dutch Influenzanet surveyed year round during the 2009 pandemic) [1] (Figure A1).

By excluding the weeks when there was no virological confirmation of influenza, we exclude the period when most ILI cases were likely due to non-influenza infections; and by considering only the number of ILI cases above the typical number of ILI cases measured in the absence of circulating viruses, one can therefore obtain a more accurate proxy of the VE against influenza. The VE calculated in this way is here designated as VE(influenza) (Table A1 and Figure 3).

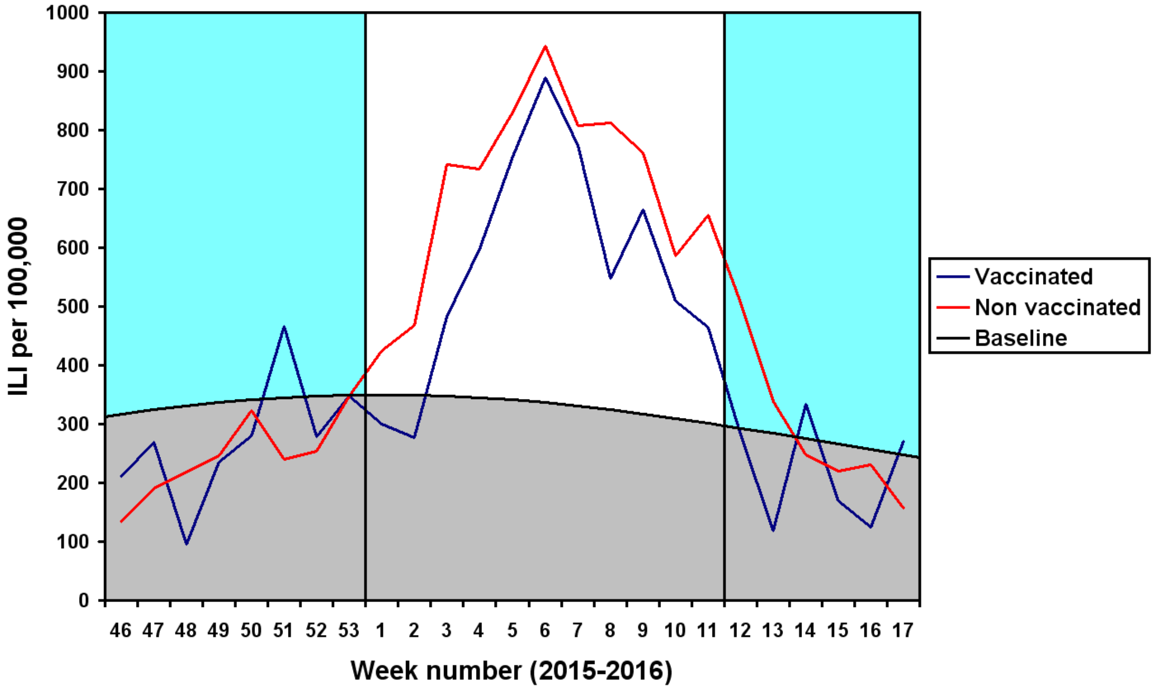

Figure A1. ILI incidence in vaccinated and unvaccinated Influenzanet participants in the Netherlands (2015-2016). *The ILI incidence was defined as the number of ILI onsets per 100,000 participant-weeks. The considered influenza period (between vertical lines: week 1 to 11 in 2016) coincides with the period that the sentinel network of medical practitioners officially proclaimed as the influenza epidemic period based on numbers of visits to the doctors and virology analyses.*

Table A1 lists the overall attack rates (AR) for ILI in vaccinated and non-vaccinated participants in 2014-15 and 2015-16, alongside with the estimated relative risk (RR) and vaccine efficacy (VE), for all participants overall, and stratified according to the presence *vs.* absence of chronic conditions and/or older age. The AR for a given period was calculated as the total number of new ILI cases in each group of participants divided by the mean number of weekly active participants in that group, during that period. The relative risk (RR) was calculated dividing the AR in vaccinated by the AR in non-vaccinated participants. The vaccine effectiveness (VE) was determined as the relative risk reduction in vaccinated participants (i.e., VE=[1-RR]x100). The 95% confidence intervals were estimated using the LaMorte Biostatistics tool.

Table A1. Estimation of influenza vaccine effectiveness (VE) in the Netherlands.

2014-2015:

Week 46 to 18	***AR vaccinated***	***AR not vaccinated***	***RR***	***P-value***	***VE (ILI) 95% CI***
All participants	474 / 3845	820 / 6626	0.996	0.943	+0.4% (-11 to +10)
60- with no chronic illness	46 / 363	567 / 4503	1.006	0.965	-0.6% (-33 to +24)
Chronic patients	410 / 3255	161 / 954	0.746	**<0.001**	**+25.4%** (+12 to +37)
60+	234 / 2521	137 / 1559	1.057	0.593	-5.7% (-29 to +14)

Week 50 to 14 (Influenza epidemic period with baseline subtracted)	***AR vaccinated***	***AR not vaccinated***	***RR***	***P-value***	***VE(influenza) 95% CI***
All participants	173 / 3963	308 / 6904	0.980	0.815	+2.0% (-17 to +18)
60- with no chronic illness	19 / 378	221 / 4709	1.072	0.769	-7.2% (-69 to +32)
Chronic patients	154 / 3350	78 / 992	0.585	**<0.001**	**+41.5%** (+24 to +65)
60+	56 / 2584	34 / 1601	1.024	0.925	-2.4% (-56 to +33)
60+ with chronic illness	56 / 2349	25 / 398	0.379	**<0.0001**	**+62.1%** (+40 to +76)
60+ with no chronic illness	0 / 235	9 / 1203	0	0.367	+100%[a]
60- with chronic illness	98 / 1001	53 / 594	1.097	0.567	-9.7% (-51 to +20)

Table A1. Estimation of influenza vaccine effectiveness (VE) in the Netherlands (cont.)

2015-2016:

Week 46 to 17	***AR vaccinated***	***AR not vaccinated***	***RR***	***P-value***	***VE (ILI) 95% CI***
All participants	418 / 4236	730 / 6208	0.839	**0.002**	**+16.1% (+6 to +25)**
60- with no chronic illness	43 /406	520 / 4175	0.850	0.275	+15% (-14 to +37)
Chronic patients	358 / 3555	111 / 833	0.755	**0.006**	**+24.5%** (+8 to +38)
60+	214 / 2845	128 / 1536	0.903	0.339	+9.7% (-11 to +27)

Week 1 to 11 (Influenza epidemic period with baseline subtracted)	***AR vaccinated***	***AR not vaccinated***	***RR***	***P-value***	***VE(influenza) 95% CI***
All participants	119 / 4353	270 / 6535	0.662	**0.0001**	**+33.8% (+18 to +46)**
60- with no chronic illness	17 / 422	203 / 4422	0.878	0.596	+12.2% (-42 to +46)
Chronic patients	100 / 3649	41 / 876	0.586	**0.003**	**+41.4%** (+16 to +59)
60+	38 / 2909	36 / 1579	0.573	**0.014**	**+42.7%** (+10 to +64)
60+ with chronic illness	36 / 2627	10 / 342	0.469	**0.029**	**+53.1%** (+6 to +77)
60+ with no chronic illness	2 / 282	26 / 1237	0.338	0.058	+66.2% (-41 to +92)
60- with chronic illness	64 / 1022	31 / 534	1.077	0.721	-7.7% (-64 to +29)

Analyses were done for all participants and also stratified by presence of chronic illness and/or age group (60-: younger than 60 years old; 60+: 60 years or older), based on self-reported influenza-like illness to Influenzanet in the Netherlands in the 2014-15 and 2015-16 seasons. AR: Attack rate; RR: Relative risk. CI: confidence interval. [a]In the "60+ with no chronic illness" stratification in 2014-15 it was not possible to calculate the 95% CI due to zero ILI cases in the vaccinated group.

References

[1] Van Noort SP: Participatory Surveillance and Mathematical Models in Epidemiologic Research: Successes and Challenges. PhD Dissertation, Universidade Nova de Lisboa, July 2014. URL: http://sander.influenzanet.eu/pdf/vnoort_thesis.pdf [accessed 2016-09-21] (Archived by WebCite® at http://www.webcitation.org/6kgeSHCnV)
